# Early and Pronounced PVR Reaction After Revitrectomy With Allogeneic Platelet Concentrate in Persistent Macular Hole

**DOI:** 10.1155/crop/5152170

**Published:** 2026-01-08

**Authors:** Patrick C. Fisel, Martin Spitzer, Aydin Özen, Christos Skevas

**Affiliations:** ^1^ Department of Ophthalmology, University Medical Center Hamburg-Eppendorf, Hamburg, Germany, uke.de

**Keywords:** case report, macular hole, platelet concentrate, proliferative vitreoretinopathy, vitrectomy

## Abstract

We report two cases of pronounced development of proliferative vitreoretinopathy (PVR) within a few days after 23G revitrectomy with allogeneic platelet concentrate (PC) in persistent macular hole in 2023–2024. None of the cases had relevant risk factors for PVR development such as intraoperative retinal tears. Because the revitrectomy with PC was performed as a no‐touch technique after a previous uncomplicated vitrectomy, it is suspected that PC was the etiological factor. Many findings from previous studies indicate that the same growth factors that are secreted by the platelets and that are thought to have a positive effect on the closure of the macular foramina were also responsible for the development of PVR. Vitrectomy with PC—usually performed with autologous PC—is considered a safe standard procedure for the treatment of large and persistent macular holes. Why such a pronounced and early PVR reaction occurred in these two cases is unclear. One possible reason could be the use of allogeneic PC in our cases. To the best of our knowledge, there are no reported cases of this kind.

## 1. Introduction

PVR is one of the most feared complications of vitreoretinal surgery, as it often leads to a chronic course of the disease and is associated with a potential severe loss of vision. Although PVR is a common complication in the surgical treatment of retinal detachment (5%–10%), it is extremely rare in vitreomacular surgery (approx. 0.05%) [1, 2]. This is due to the fact that pathophysiologically, PVR usually occurs on the floor of a larger vitreoretinal break, through which pigment epithelium (PE) and retinal cells can migrate into the vitreous and thus come into contact with various growth factors [3]. Mostly PVR appears in average within a time interval of 1–2 months after vitreoretinal surgery [4–6]. An early PVR reaction within 1 week, even after surgery of retinal detachment, is rather rare at 13.6% [5].

Vitrectomy, usually with autologous platelet concentrate, is used as a secondary procedure for persistent macular holes after primary unsuccessful pars plana vitrectomy (PPV) with peeling of the internal limiting membrane (ILM) [7]. This type of surgery is particularly gentle and can be regarded as a no‐touch technique, as a PPV with ILM peeling has already been performed.

A total of eight re‐vitrectomies with allogeneic PC were performed at our clinic in 2023–2024 for persistent macular holes, of which seven were done with C2F6 and one with silicone oil endotamponade. We report two cases in this period of a very early PVR reaction within 1 week after surgery. To our best knowledge, there are no reported cases of this kind.

## 2. Case 1

**Figure 1:**
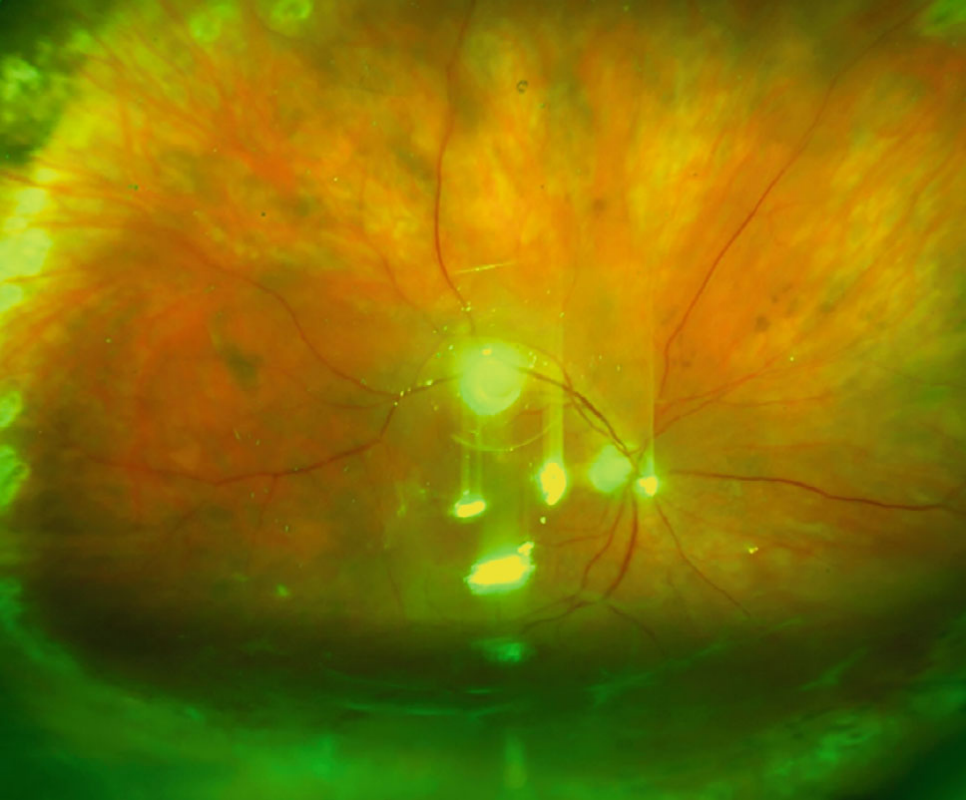
Preoperative view of the fundus with inferior tractional retinal detachment with PVR.

**Figure 2:**
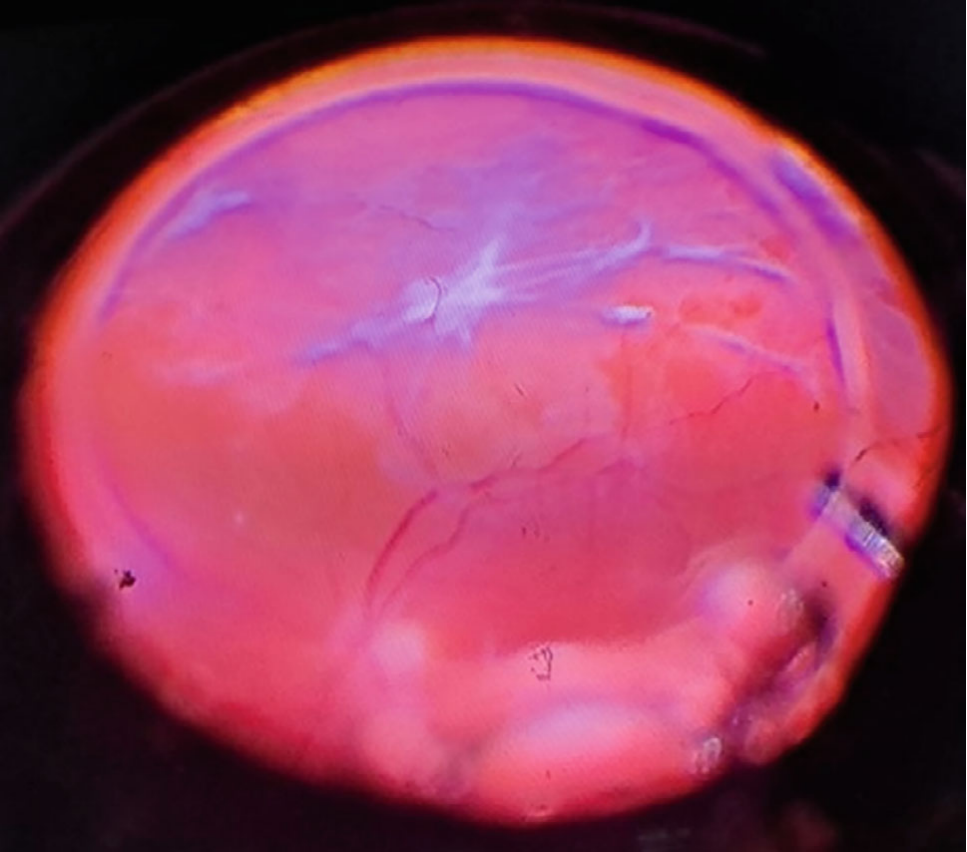
Intraoperative view of the fundus with inferior PVR membranes.

**Figure 3:**
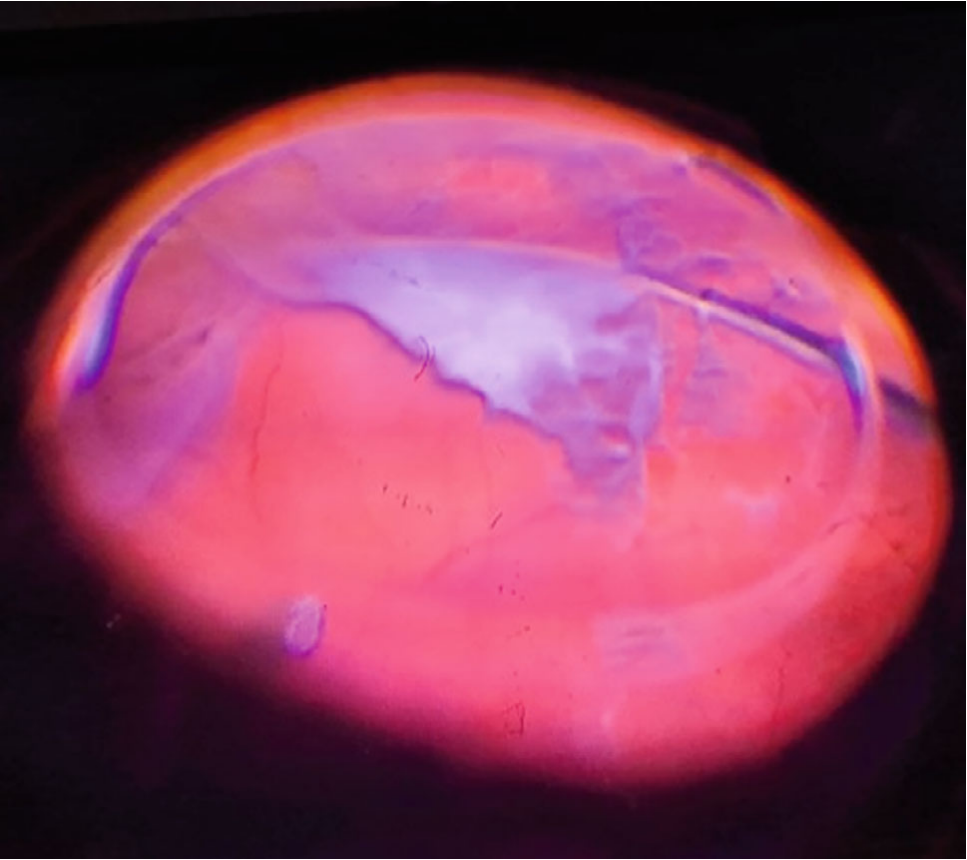
Intraoperative view of the fundus shows the PVR membrane peeling.

Case 1 is a 68‐year‐old man presented with 4 months of metamorphopsia and visual deterioration in the right eye with medium‐sized macular hole (290 *μ*m) in vitreomacular traction syndrome (VMT) and epiretinal gliosis (ERM). The hole‐edge configuration was with a cuff and edematous cysts relating to CLOSE classification [8]. In addition, there was a corticonuclear cataract. The visual acuity at first presentation was 6/30. We decided to perform a combined phacovitrectomy with membrane peeling (MP) and ILM peeling. Due to the patient′s health reasons, the surgery was performed 2 months later and was done with intraocular gas endotamponade (C3F8) and an endolaser retinopexy of peripheral retinal degenerations. Two months after surgery, the patient presented to our outpatient clinic with persistent macular hole (345 *μ*m) and visual acuity of 6/60, so we arranged a revitrectomy with allogeneic platelet concentrate, which was performed 1 week later. Intraoperatively, 1–2 drops of PC were installed and a gas endotamponade (C2F6) was performed. Postoperative positioning was supine on the day of surgery. After the day of surgery, no further positioning was ordered. On the second postoperative day, an inferior retinal detachment was detected during the ward round. There was also significant inflammation in the anterior chamber with Grade 2+ according to the SUN classification [9]. No retinal tears could be detected after examination by a junior doctor and two different senior doctors, which is why an exudative amotio was suspected and the patient was called in again 2 days later. On re‐presentation, an inferior tractional retinal detachment with PVR and three retinal tears in the surrounding retinal mid‐periphery were found, so that a revitrectomy with peeling of the PVR and renewed injection of PC and 5000 cSt silicone oil was performed on the same day (Figures 1, 2, and 3). The postoperative visual acuity was still 6/60. Two months later, at the follow‐up visit, the retinal findings under oil were stable with a visual acuity of 6/15.

A 360° inspection of the peripheral retina under indentation was performed in each of the surgeries. Neither in the PPV with MP and ILM peeling, in the PPV with PC, nor in the postoperative visits in the first 2 days after surgery were retinal tears detectable. Intraoperative endolaser retinopexy was performed for peripheral retinal degenerations and a complete vitrectomy with peripheral vitreous removal with vitreous base shaving was done. Apart from prior intraocular surgery, none of the known intraoperative (retinal tears, retinal or choroidal detachment, bleeding, cryogenic application, use of air, or sulfur hexafluoride) or patient‐associated (positive history for smoking or uveitis, young age, or aphakia) risk factors for PVR were present [3].

## 3. Case 2

**Figure 4:**
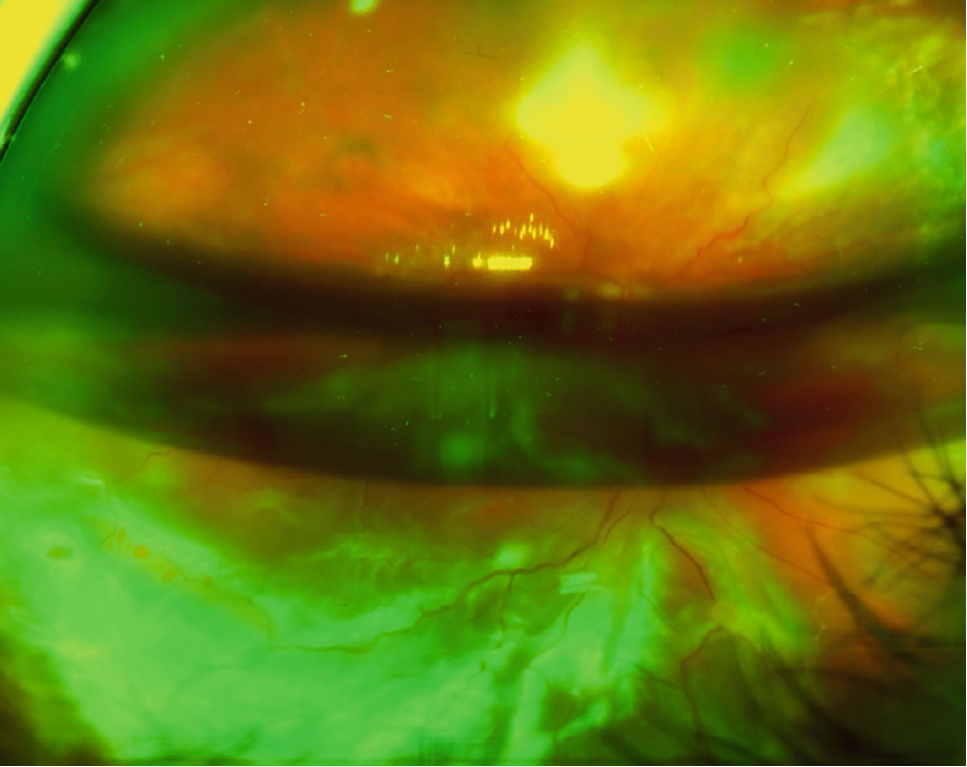
Wide field fundus photography shows an inferior retinal detachment due to intensive PVR.

Case 2 is a 74‐year‐old woman presented to our outpatient clinic with a 3 to 4‐month history of deteriorating visual acuity with a right‐sided medium‐sized macular hole (260 *μ*m, flat edges, no cysts, and VMT or ERM) and corticonuclear cataract. The visual acuity was 6/30. We performed 1 week later a combined phacovitrectomy with ILM peeling, endolaser application in the presence of peripheral retinal degeneration and gas endotamponade (C2F6). On re‐presentation, 1 month later there was a persistent macular hole without change of the visual acuity, so the decision was made to perform a revitrectomy and application of allogeneic PC, which was performed 1 week later. Postoperative positioning was supine on the day of surgery. After the day of surgery, no further positioning was ordered. Postoperatively, there was significant fibrin reaction of the anterior chamber, so that funduscopy could not be performed and intensive treatment with dexamethasone eye drops was started. Six days after surgery, the patient presented to us as an emergency case with inferior retinal detachment with macular involvement and massive PVR reaction (Figure 4). The visual acuity was hand movements. The following day, a revitrectomy with endolaser application and 5000 cSt silicone oil endotamponade was performed. Intraoperatively, five retinal tears at the inferior temporal vascular arcade in the retinal mid‐periphery and an inferior PVR with pronounced intraretinal PVR strands were detected, so that retinal reattachment could only be achieved after inferior retinectomy.

As in Case 1, an intraoperative 360° inspection of the peripheral retina under indentation was performed in all surgeries and no retinal tears were visible. Additionally, a complete vitrectomy with intraoperative laser application for retinal degeneration was performed. No further risk factors for PVR were present in this case.

## 4. Discussion

We report two cases of very early PVR development within a few days after PPV with PC for persistent macular hole. The surgeries were performed by two different retinal surgeons, both with expert level. In none of the operations, including the previous vitrectomies, was a vitreoretinal break visible, which is considered to be the pathophysiological basis for PVR [3]. Retinal tears could only be detected later on in the area of the PVR in the mid‐periphery. PVR is a complication especially after retinal detachment and occurs in about 5%–10% of its surgical treatment but only in 13.6% of cases within the first postoperative week [1, 5]. In vitreomacular surgery, it is much rarer, at approximately 0.05% [2]. Vitrectomy with PC as a secondary procedure for persistent macular holes can be regarded as a no‐touch surgery, as vitrectomy was already performed within the first surgery. It is, therefore, all the more surprising that such an early PVR reaction occurred in these cases.

Common to both cases was that the initial surgical treatment of the macular break was performed very late (4–6 months) after the onset of symptoms due to delayed presentation of the patients in our outpatient department. The patients were 68 and 74 years old. In both cases, an intense postoperative inflammatory reaction of the anterior chamber was visible, in Case 2 even with fibrin formation. Anamnestically, no rheumatological or other chronic inflammatory diseases were known. The drug history was unremarkable. Because there was no PVR reaction in any of the initial vitrectomies with ILM peeling, but an intense and early PVR formation occurred after PPV with PC without any significant irritation of the retina apart from the PC application, it can be assumed that the PVR formation is related to the PC. Previous publications on the use of platelet concentrates in vitreomacular surgery all report on the use of autologous concentrates. For logistical reasons, our clinic used irradiated platelet concentrate produced by apheresis from the plasma of a single donor. Compatibility with AB0 has been checked beforehand. The PC was prepared in accordance with German guidelines for the collection of blood and blood components at the Institute for Transfusion Medicine of our clinic. Contamination was ruled out. The PCs belonged to different series. Severe transfusion reactions associated with the use of platelet concentrates are considered very rare side effects. However, since it is also known that the eye has ocular immune privilege, it cannot be said with certainty whether this can also be applied to intraocular applications. An immunogenic reaction to these foreign blood products could be a possible explanation for the early PVR reaction.

Intraoperative or postoperative hemorrhage in vitrectomy is also considered to be a risk factor for PVR and a formation of PVR can also occur weeks to months after persistent, untreated intravitreal hemorrhage [1, 10]. The use of various blood products has been reported in the surgical treatment of macular holes. Whole blood (WB), fibrin rich plasma (FRP) and PC are used [7]. The first contains erythrocytes and leukocytes and appears to be less effective for the morphological closure of a macular hole than PC, at least without additional MP [11]. FRP is produced as a centrifugate from WB and contains platelets in addition to plasma with coagulation factors such as thrombin. PC can be regarded as a further centrifugate from FRP and contains an increased proportion of platelets. The positive effect on the closure of macular holes is much discussed and is attributed to the influence of thrombin and in particular the growth factors contained in the alpha‐granules of the platelets, such as platelet derived growth factor (PDGF), basic fibroblast growth factor (b‐FGF), transforming growth factor‐*β* (TGF‐*β*), vascular endothelial growth factor (VEGF), epithelial growth factor (EGF) and angiopoietin 1 (ANG‐1) [12–16]. It is postulated that the surgical procedure with ILM peeling activates Müller glial cells, as well as basement membrane components and the secretion of collagen, and that the growth factors additionally stimulate these processes [17, 18].

Interestingly, it is the same growth factors and the same healing effects that are utilized in the closure of macular holes that are considered to be the pathophysiological cause of PVR: the stimulation of Müller and other glial cells as well as PE cells, cell migration, upregulation of inflammation, production of extracellular matrix, and proliferation and contraction of migrated fibroblastic cells [3, 19–22]. PDGF in particular is considered to play a key pathophysiological role in the formation of PVR [23, 24]. In animal models, no PVR response could be induced in rabbit eyes, which underwent vitrectomy by the installation of PDGF receptor knock‐out fibroblasts. However, when the fibroblasts carried the PDGF receptor, 80% of the eyes showed a PVR response [23]. In porcine eyes, the installation of PC after partial vitrectomy with cryogenic application and retinotomy was also shown to significantly increase PVR formation [25]. In the porcine eye, which is anatomically very similar to the human eye [26], the application of 0.2 mL PC triggered a pronounced PVR within the first week, as in our reported cases. The amount of PVR response provoked depended on the amount of platelets injected. However, the amount of 1–2 drops injected by us corresponds to only 0.05 mL and is just a quarter of the amount used in the animal model.

Vitrectomy with autologous PC is a widely used method for the treatment of persistent macular holes and is considered effective and very safe, as it is a no‐touch technique. To the best of our knowledge, there are no reported cases of PVR for these procedures. These two cases show that, without significant risk factors for PVR, a strong and early PVR reaction can occur. Although the pathophysiological cause remains unclear, an aetiological link with the PC seems likely. One explanation could be the use of allogeneic PC in our cases.

## Ethics Statement

No written consent has been obtained from the patients as there is no patient identifiable data included in this case reports.

## Conflicts of Interest

The authors declare no conflicts of interest.

## Funding

Open Access funding enabled and organized by Projekt DEAL.

## Data Availability

The data that support the findings of this study are available from the corresponding author upon reasonable request.
